# Numerical Study of Magnetoacoustic Signal Generation with Magnetic Induction Based on Inhomogeneous Conductivity Anisotropy

**DOI:** 10.1155/2013/161357

**Published:** 2013-03-26

**Authors:** Xun Li, Sanqing Hu, Lihua Li, Shanan Zhu

**Affiliations:** ^1^College of Computer Science, Hangzhou Dianzi University, Hangzhou 310018, China; ^2^College of Life Information Science and Instrument Engineering, Hangzhou Dianzi University, Hangzhou 310018, China; ^3^College of Electrical Engineering, Zhejiang University, Hangzhou 310027, China

## Abstract

Magnetoacoustic tomography with magnetic induction (MAT-MI) is a noninvasive imaging modality for generating electrical conductivity images of biological tissues with high spatial resolution. In this paper, we create a numerical model, including a permanent magnet, a coil, and a two-layer coaxial cylinder with anisotropic electrical conductivities, for the MAT-MI forward problem. We analyze the MAT-MI sources in two cases, on a thin conductive boundary layer and in a homogeneous medium, and then develop a feasible numerical approach to solve the MAT-MI sound source densities in the anisotropic conductive model based on finite element analysis of electromagnetic field. Using the numerical finite element method, we then investigate the magnetoacoustic effect of anisotropic conductivity under the inhomogeneous static magnetic field and inhomogeneous magnetic field, quantitatively compute the boundary source densities in the conductive model, and calculate the sound pressure. The anisotropic conductivity contributes to the distribution of the eddy current density, Lorentz force density, and acoustic signal. The proposed models and approaches provide a more realistic simulation environment for MAT-MI.

## 1. Introduction

Since Henderson and Webster reported an impedance camera to generate the electrical impedance image of the thorax [1], it is of increasing interests to noninvasively measure the electrical impedance of biological tissues. Several approaches, such as electrical impedance tomography (EIT) [2, 3], magnetic induction tomography (MIT) [4, 5], magnetic resonance EIT (MREIT) [6], magnetoacoustic tomography (MAT) [7, 8], and Hall effect imaging (HEI) [9], have been developed to image the electrical impedance distribution. Among these technologies, EIT, MREIT, and MAT/HEI inject electrical currents into the imaging object through the surface electrodes, so that they have to face the “shield effect” [10, 11] caused by a low-conductivity tissue layer surrounding the object and therefore have difficulties in imaging the electrical impedance of deep biological tissue with high spatial resolution. MIT excites the deep biological tissue with time-variant magnetic field and measures the secondary magnetic field produced by the eddy current to reconstruct electrical impedance images. However, the inverse problem in MIT, as in EIT, is an ill-posed problem. 

 Magnetoacoustic tomography with magnetic induction (MAT-MI) is a newly proposed electrical impedance imaging modality [11]. In MAT-MI, an object is placed in an external static magnetic field **B**_0_ and a time-variant magnetic field **B**_1_ to induce the eddy currents **J** in the object. The eddy currents are subject to Lorentz forces to induce sound vibrations in the object. The emitted sound signals are detected around the object to reconstruct the electrical impedance images of the imaging object. Through combining magnetism and sonography, MAT-MI can excite deep tissues and image the electrical impedance with high spatial resolution. As a result of the sound measurement around the specimen, MAT-MI has a well-posed inverse problem.

Similar to MAT/HEI, MAT-MI is based on the Lorentz force-induced vibrations. The difference among them is that MAT-MI uses time-variant magnetic field, while the MAT/HEI applies current injection, and therefore the MAT/HEI sound sources are only at the boundary between regions of differing conductivity for a piecewise homogeneous isotropic conductor [7], while those of MAT-MI exist everywhere in the conductor. 

 It is well known that some biological materials, such as bone and skeletal muscle, are distinctly anisotropic [12]. Recently, several studies have been developed to explore the effect of electrical anisotropy, such as the influence of white matter anisotropy on EEG source localization [13], inhomogeneous anisotropic cardiac tissues [14], and the effect of conductivity anisotropy on EIT [15]. Another study has reported that the diffusion anisotropy in breast cancer is significantly different from that in normal tissue [16]. The water diffusion may have a relation with the electrical conductivity in a tissue, and the conductivity tensor can be obtained from the diffusion tensor [17]. It is obvious that breast cancers may have different anisotropic conductivity tensor from that of normal tissues.

 In previous works, there are many theories and simulation models, as shown in Table 1, to study MAT-MI principles. 

 In the present study, we analyze the MAT-MI sound source densities in a homogeneous conducting medium and on a thin conductive boundary layer and build a magnet and a circular coil to produce inhomogeneous static magnetic field and time-variant magnetic field. We create a two-layer coaxial cylinder with different anisotropic conductivity values and solve the MAT-MI forward problem with the aid of the finite element method (FEM). By comparing anisotropic conductive model with isotropic conductive model, we investigate the magnetoacoustic effect of the conductivity anisotropy. We also discuss the difference of sound signal generation between MAT-MI and MAT/HEI.

## 2. Theory

According to the previous works [7, 11], MAT-MI wave equation can be described as

(1) $$\begin{array}{c} \nabla^{2}p-\frac{1}{c_{s}^{2}}\frac{\partial^{2}p}{\partial t^{2}}=\nabla \cdot \left(J\times B\right), \end{array}$$

where *p* is the acoustic pressure, *c*_*s*_ is the sound speed, **J** is the induced eddy current density, and **B** is the magnetic flux density including the static magnetic flux density **B**_0_ and the time varying magnetic flux density **B**_1_. The cross product of **J** and **B** is the Lorentz force density, and the divergence of the Lorentz force density is the sound source density. Here, we study the sound source density on three conditions including homogeneous isotropic conducting medium, a thin conductive boundary layer in the heterogeneous conducting medium, and anisotropic conducting medium.

### 2.1. Homogeneous Isotropic Conducting Medium

In this case, the conductivity *σ* is a constant in solving domain. We assume that the electrical currents producing the time-variant magnetic field are outside of the imaging object, so that the curl of **B** is zero [7]. Then, we have [21]

(2) ∇·(J×B)=(∇×J)·B−J·(∇×B)=(∇×J)·B=(∇×σE)·B=−σ∂B∂t·B.

Since the static magnetic field is time invariant, the sound source density is

(3) $$\begin{array}{c} \nabla \cdot \left(J\times B\right)=-\sigma \frac{\partial B_{1}}{\partial t}\cdot B. \end{array}$$

### 2.2. A Thin Conductive Boundary Layer in a Heterogeneous Conducting Medium

When the conductivities are not homogeneous but changed abruptly, the eddy current densities and the corresponding Lorentz force densities are not continuous on both sides of the boundary layer. The source term, which is the divergence of the Lorentz force density, should be calculated in a different way. To solve the MAT-MI sources, we assume a very small tube on the boundary layer and apply the Gauss theorem on the source term to avoid the divergence on the jump discontinuity. 

**Figure 1:**
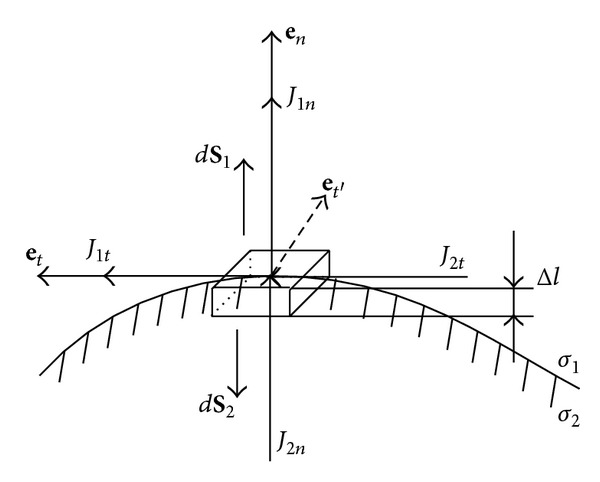
A tube on the boundary between two homogeneous conducting media.

As shown in Figure 1, we consider a small tube on the boundary layer between two homogeneous isotropic media with conductivity values of *σ*_1_ and *σ*_2_. **e**_*n*_ is the outward normal to the tangent plane, *S*_1_ and *S*_2_ are the two surfaces of the tube, and the outward normal to the *S*_1_ and *S*_2_ is, respectively, in the same and opposite directions as **e**_*n*_. *J*_1*n*_, *J*_2*n*_ and *J*_1*t*_, *J*_2*t*_ are, respectively, the normal and tangential components of the eddy current densities, **e**_*t*_ is in the same direction as *J*_1*t*_ and *J*_2*t*_, and e_*t*′_ is orthogonal to both *J*_1*t*_ and *J*_1*n*_. The thickness of the tube Δ*l* is assumed to be infinitesimal.

Based on electromagnetic theory, the electromagnetic field boundary conditions are as follows [25]:

(4) $$\begin{array}{c} J_{1n}=J_{2n},\;\;E_{1t}=E_{2t}. \end{array}$$

Due to the magnetic field continuity across the boundary, we have

(5) $$\begin{array}{c} B_{1}=B_{2}=B, \end{array}$$

where **B**_1_ and **B**_2_ are the magnetic field on both sides of the boundary layer.

 Thus, the Lorentz force density, the cross product of the eddy current density **J** and magnetic flux density **B** on the boundary layer, can be written in the orthogonal coordinates system (**e**_*n*_, **e**_*t*_, **e**_*t*′_) as follows:

(6) $$\begin{array}{c} J\times B=\left|\begin{array}{ccc} n & t & t^{\prime }\\ J_{n} & J_{t} & 0\\ B_{n} & B_{t} & B_{t^{\prime }} \end{array}\right|=J_{t}B_{t^{\prime }}n-J_{n}B_{t^{\prime }}t+\left(J_{n}B_{t}-J_{t}B_{n}\right)t^{\prime }. \end{array}$$

Applying the Gauss theorem, we have

(7) $$\begin{array}{c} \int_{V}\nabla \cdot \left(J\times B\right)dV=\oint_{S}\left(J\times B\right)dS. \end{array}$$

In the case of the small tube as shown in Figure 1, we have Δ*l* → 0 and

(8) ∮S(J×B)dS=∫S1J1tBt′n·dS1+∫S2J2tBt′n·dS2=(σ1−σ2)E1tBt′S1,

where  **e**_*t*′_  is in the same direction as  **e**_*t*_ × **e**_*n*__._

 Then, we have the sound source of the small tube on the boundary layer as follows:

(9) $$\begin{array}{c} \int_{V}\nabla \cdot \left(J\times B\right)dV=\left(\sigma_{1}-\sigma_{2}\right)E_{1t}B_{t^{\prime }}S_{1}. \end{array}$$

From formula (9), we can compute the sound sources on the boundary layer through the outward normal to the boundary surface, the intensity, and direction of the **E** and **B**. Zhou et al. got the same result as formula (9) [24].

### 2.3. A Thin Conductive Boundary Layer in the Anisotropic Conducting Medium

Considering the MAT-MI sound source in a homogenous anisotropic conducting medium, the conductivity value *σ* is not a constant but a tensor. We have

(10) $$\begin{array}{c} \nabla \cdot \left(J\times B\right)=\nabla \cdot \left\{\left(\left[\begin{array}{ccc} \sigma_{xx} & \sigma_{xy} & \sigma_{xz}\\ \sigma_{yx} & \sigma_{yy} & \sigma_{yz}\\ \sigma_{zx} & \sigma_{zy} & \sigma_{zz} \end{array}\right]\left[\begin{array}{c} E_{x}\\ E_{y}\\ E_{z} \end{array}\right]\right)\times B\right\}. \end{array}$$

We will introduce a numerical solution of this case by using finite element interpolation in Section 3.3.1.

On the boundary layer, the electromagnetic field boundary conditions are the same as described in formula (4), and we can use the same method as illustrated in Section 2.2 to analyze the MAT-MI sound source on the boundary layer in an anisotropic heterogeneous conducting medium. Then, we have

(11) ∫V∇·(J×B)dV  =Bt′S1(([σ1xxσ1xyσ1xzσ1yxσ1yyσ1yzσ1zxσ1zyσ1zz]          −[σ2xxσ2xyσ2xzσ2yxσ2yyσ2yzσ2zxσ2zyσ2zz])[E1txE1tyE1tz])·et,

where *E*_1*tx*_, *E*_1*ty*_, and *E*_1*tx*_ are the decomposition of the tangential component of the electric field in the Cartesian coordinate system.

### 2.4. Solution of the Wave Equation

Applying Green's function, we have the solution of the MAT-MI wave equation in free space [11] as follows:

(12) p(r,t)=−14π∮Vdr′ ×∫−∞∞∇r′·(J(r′,t′)×B(r′,t′))δ(t′−t+|r−r′|/cs)|r−r′|dt′,

where *V* is the source domain, **r**′ is a sound point source, **r** is a point detecting the sound radiation from the sound point sources, *t* is the time to detect the sound signal, and *δ* is a Dirac Delta function. Using the previous formula, we can compute the sound pressure radiated out from the sources.

## 3. Simulation Study

In the previous studies, numerical simulations were conducted on solid models with isotropic conductivity [21, 23, 24] or a uniform sheet with homogeneous conductivity anisotropy [20], under homogeneous static magnetic field. In the present study, we adopt a permanent magnet to produce a more realistic and complex inhomogeneous static magnetic field and conduct numerical simulations on a conductor with inhomogeneous anisotropic conductivities. Based upon the present models, we perform the electromagnetic analysis using the finite element method software ANSYS and calculate the sound source densities in the homogeneous anisotropic medium on the conductivity boundary and the spatial distribution of the acoustic pressure. Finally, we compare the eddy currents, Lorentz forces, MAT-MI sources, and sound pressure of the anisotropic and isotropic conductive models to investigate the effect of conductivity anisotropy on the MAT-MI signal generation.

### 3.1. Model

**Figure 2:**
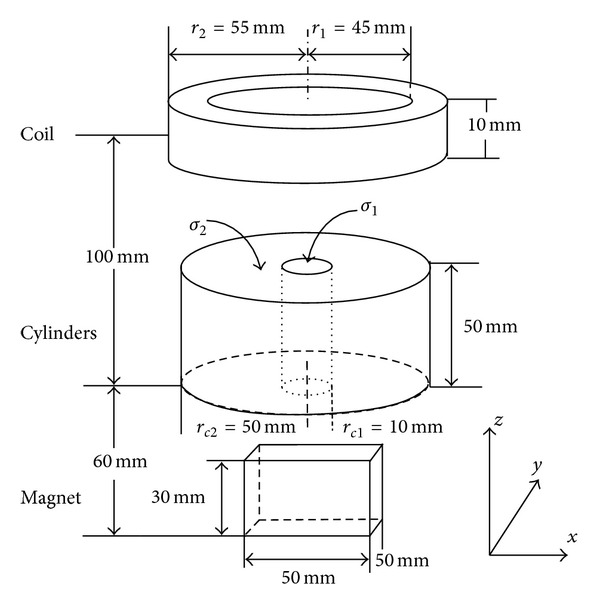
Model geometry.

The current simulation models include a two-layer conductive cylinder, a magnet, a real shape coil, and the surrounding water and air media.  Figure 2 shows the coaxial cylindrical model together with the coil and magnet models. In the conductive models, the radii of the inner and outer layer cylinders are, respectively, 10 and 50 mm, and the anisotropic conductivity values of the inner and outer layers are, respectively, *σ*_1_ = [*σ*_1*x*_ = 0.25, *σ*_1*y*_ = 0.125, *σ*_1*z*_ = 0.2] and *σ*_2_ = [*σ*_2*x*_ = 0.04, *σ*_2*y*_ = 0.1, *σ*_2*z*_ = 0.2] S/m. The conductivity of the two layers in the *x* direction *σ*_1*x*_ and *σ*_2*x*_ is the same as the isotropic conductivity values adopted in the previous work [19], and the difference between *σ*_1*y*_ and *σ*_2*y*_ is much smaller than that between *σ*_1*x*_ and *σ*_2*x*_. This allows us to investigate the change of the eddy current density caused by the electrical anisotropy through comparing with those induced in the isotropic conductive models. The cylindrical shape of the conductor is similar to the columnar phantom employed in previous MAT-MI experiments [19, 26–28]. The width, length, and height of the permanent magnet are, respectively, 50, 50, and 30 mm. The coil has a height of 10 mm, with an inner radius and an outer radius of 45 mm and 55 mm, respectively. The water surrounding the conductive model is a cylinder with a radius of 80 mm and a height of 70 mm. The conductivity value of the water *σ*_*w*_ is 0.4 S/m. The coil, magnet, and water are surrounded by the cylindrical air medium with a radius of 200 mm and a height of 300 mm.

**Figure 3:**
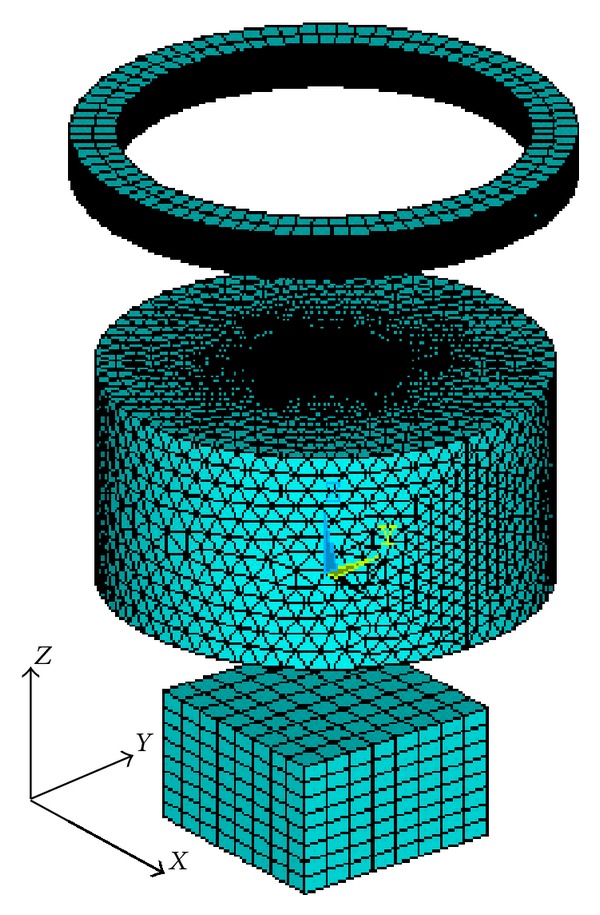
Meshed grids of the coil, permanent magnet, and two-layer coaxial cylindrical conductive models.

Taking the center of the underside of the two-layer coaxial cylinder as the origin, the bottom of the coil is 95 mm above, and the top of the magnet is 30 mm below the origin. The top and bottom of the water are, respectively, 60 mm above and 10 mm below the origin. The coil, two-layer coaxial cylinder, and water and air models are symmetric with respect to the *z*-axis, and the magnet is symmetric about the plane *x* = 0 and *y* = 0. The symmetry of the solid model allows us to explore the effect of the electrical anisotropy specifically. The injected currents into the coil obey a quasi-step function described as

(13) $$\begin{array}{c} J\left(t\right)=\{\begin{array}{cc} J_{\text{end}} & t\geq T_{\text{end}},\\ \frac{\left(t-T_{\text{start}}\right)*J_{\text{end}}}{T_{\text{end}}-T_{\text{start}}} & T_{\text{start}}<t<T_{\text{end}},\\ 0 & t\leq T_{\text{start}}, \end{array} \end{array}$$

where *T*_start_ and *T*_end_ are the time to begin and finish the electrical current injection, *T*_end_ − *T*_start_ = 5*E* − 6 s, and *J*_end_ = 3*E* + 8 A/m^2^ is the magnitude of the current density at the end of the current injection. The sound speed is assumed to be homogeneous and isotropic in all the media and is set to be 1500 m/s. We create the solid models in ANSYS, meshed the coil and magnet with hexahedrons, and meshed the conductive cylinders, water, and air media with tetrahedrons. The meshed grids of the coil, magnet, and two-layer coaxial cylindrical conductive models are shown in Figure 3. 

In order to evaluate the impact of the conductivity anisotropy on the magnetoacoustic signals, we adopt an isotropic conductive model sharing the same geometry with the aforementioned model. The isotropic conductivity values of the inner and outer cylinders are, respectively, 0.25 and 0.04 S/m, which are the same as those adopted in the previous isotropic conductive model [19]. 

### 3.2. Procedure

1. Performing the FEM electromagnetic analysis, obtaining the Lorentz force density **F** on every node, eddy current density **J** and magnetic flux density **B** on every element. And extracting the elements neighboring to the boundary layer and the corresponding nodes of the elements on the boundary layer.
2. Calculating the sound source density in the finite elements in the homogeneous medium and in the small tubes on the conductivity boundary.
3. Regarding the sound sources in the elements and tubes as point sources, assuming the sound speed in the solving domain is a constant *c*_*s*_ = 1500 m/s, and applying the discrete form of formula (12) to calculate the sound field as follows:

(14) $$\begin{array}{c} P\left(r,t\right)=-\frac{1}{4\pi }\sum_{j=1}^{M}\frac{\nabla \cdot \left(J_{j}\left(r_{j}^{\prime },t-R/c_{s}\right)\times B_{j}\left(r_{j}^{\prime },t-R/c_{s}\right)\right)}{R}, \end{array}$$

where *M* is the number of the point sources, *R* = |**r** − **r**′|.

### 3.3. Sound Source

#### 3.3.1. Source in the Homogeneous Medium

For the MAT-MI source density in the homogeneous anisotropic conducting medium, we have

(15) $$\begin{array}{c} \nabla \cdot \left(J\times B\right)=\nabla \cdot F=\frac{\partial F_{x}}{\partial x}+\frac{\partial F_{y}}{\partial y}+\frac{\partial F_{z}}{\partial z}. \end{array}$$

After performing the finite element analysis of electromagnetic field, we have the nodal solutions of the Lorentz force density. We apply the FEM interpolation to the Lorentz force density in each element to count the sound source density.

**Figure 4:**
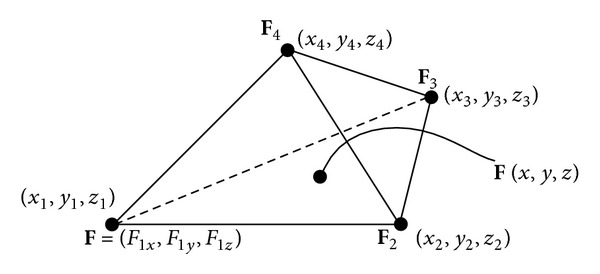
The nodal solutions of the Lorentz force density in a tetrahedral element.

 As shown in Figure 4, in a first-order tetrahedral element, **F**_1_, **F**_2_, **F**_3_, and **F**_4_ are the nodal solutions of the Lorentz force density, and **F**(*x*, *y*, *z*) is the Lorentz force density on a point in an element. Using the finite element interpolation [29], we have

(16) $$\begin{array}{c} F\left(x,y,z\right)=a^{e}+b^{e}x+c^{e}y+d^{e}z, \end{array}$$

where **a** ^*e*^, **b** ^*e*^, **c** ^*e*^, and **d** ^*e*^ are vectors as **a** ^*e*^ = [*a*_*x*_^*e*^, *a*_*y*_^*e*^, *a*_*z*_^*e*^] and so on. All the vectors are determined by the coordinates of the four tetrahedral vertices and the nodal solutions of the Lorentz force density **F**. Substituting (16) into (15), we have

(17) ∇·(J×B)=∇·F=bxe+cye+dze=16Ve(∑j=14bjeFxj+∑k=14cjeFyj+∑j=14djeFzj),

where *F*_*xj*_, *F*_*yj*_, and *F*_*zj*_ are the three Cartesian components of the nodal values of the Lorentz force and *b*_*j*_^*e*^, *c*_*j*_^*e*^, and *d*_*j*_^*e*^ are the coefficients determined from the expansion of the determinants of the elemental interpolation [29]. Through computing formula (17), we can analyze the MAT-MI sound source density in a homogeneous anisotropic conducting medium based on the solutions of the finite element analysis.

#### 3.3.2. Source on the Conductivity Boundary

**Figure 5:**
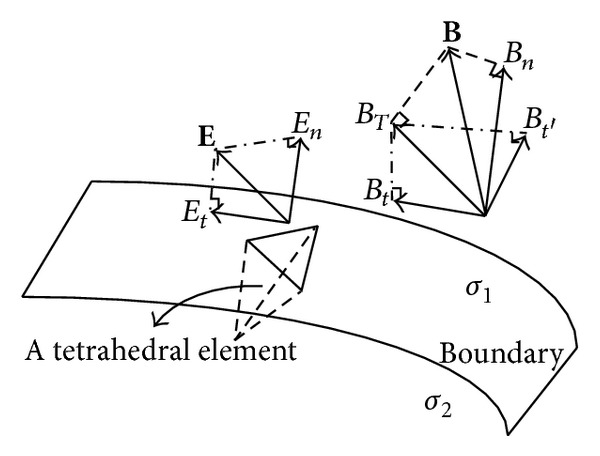
Orthogonal decomposition of **E** and **B** on the boundary surface.

Considering a tiny tetrahedral element neighboring to the boundary, we apply formula (11) to solve the MAT-MI sound source on the conductivity boundary. Since the numerical solutions of the electromagnetic analysis satisfies the boundary conditions as described in formulas (4) and (5), we assume that the electric field **E** and magnetic flux density **B** in the tetrahedral element which have three nodes adhering to the boundary are closely approximate to those of the points on the boundary surface. And then, we adopt the elemental solutions of the **E** and **B** to compute the sound sources on the boundary and decompose them, as shown in Figure 5, to compute the *E*_*t*_ and *B*_*t*′_. The procedure is as follows:

1. Extracting three nodes of the element on the boundary layer and computing the area of the triangle *S* and the outward normal **e**_*n*_.
2. Decomposing the **B** into the orthogonal components *B*_*n*_ and **B**_*T*_, where the **B**_*T*_ is the projection of the **B** on the tangent plane.
3. Mapping the **E** onto the **e**_*n*_ and computing the *E*_*n*_, *E*_*t*_, and **e**_*t*_.
4. Orthogonally decomposing **B**_*T*_ into the *B*_*t*_ and *B*_*t*′_.
5. Calculating the sound sources in accordance with formula (11).

## 4. Results

**Figure 6:**
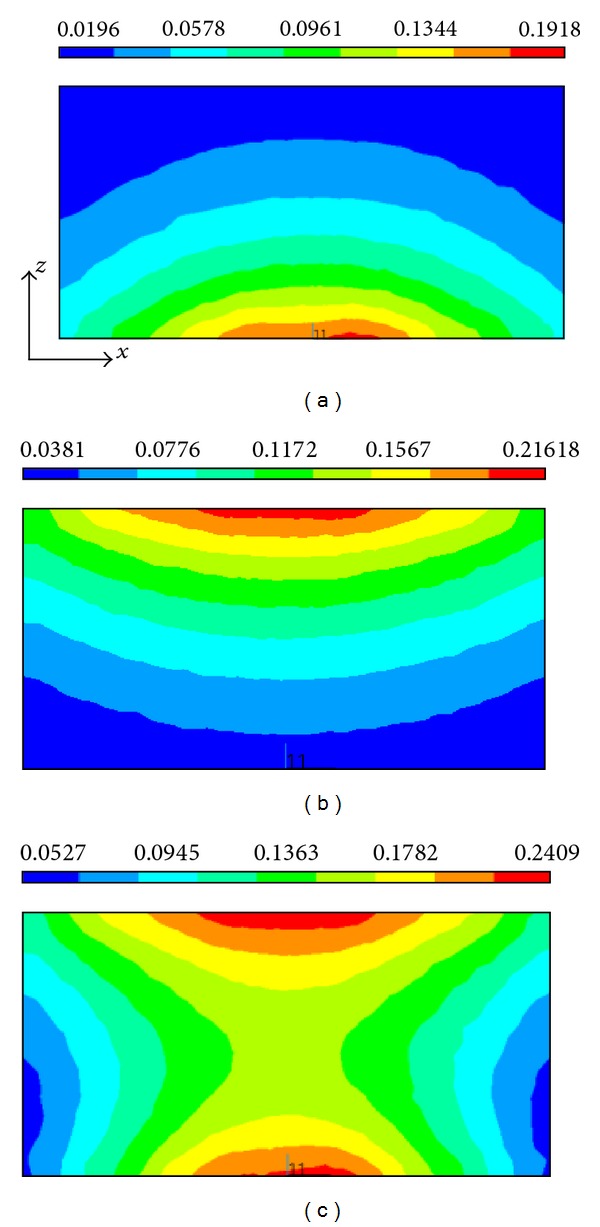
Magnetic flux density in the coaxial cylinder produced, respectively, by (a) magnet, (b) coil, and (c) both magnet and coil. The unit of magnetic flux density is Tesla (T).

**Figure 7:**
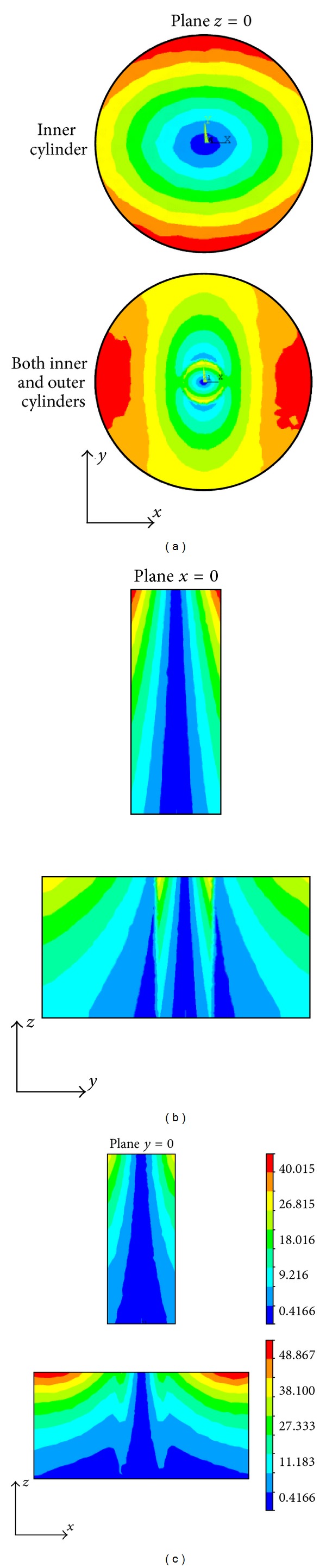
The distribution of eddy current density in the inner and outer cylinders on the planes *z* = 0, *x* = 0, and *y* = 0. The unit of eddy current density is Ampere/m^2^ (A/m^2^).

In this simulation study, we employ a real shape coil and a permanent magnet to produce the inhomogeneous magnetic field and static magnetic field and perform an electromagnetic field finite element analysis on the conductive models with electrical anisotropy. The numerical simulations are performed in SI system (international systems of units), and the units of the magnetic flux density, eddy current density, Lorentz force density, and sound pressure are, respectively, Tesla (T), Ampere/m^2^ (A/m^2^), Newton/m^3^ (N/m^3^), and Pascal (Pa). The inhomogeneous magnetic flux density produced, respectively, by the coil, the magnet, and both the coil and magnet is shown in Figure 6. The distribution of the eddy current density in the inner cylinder and both the inner and outer cylinders is illustrated in the *x* = 0, *y* = 0, and *z* = 0 planes. From Figure 7, it is obvious that the distribution of the eddy current density strongly respond to the anisotropic conductivity so as to cause an apparent aberration in the *x- y* plane.

**Figure 8:**
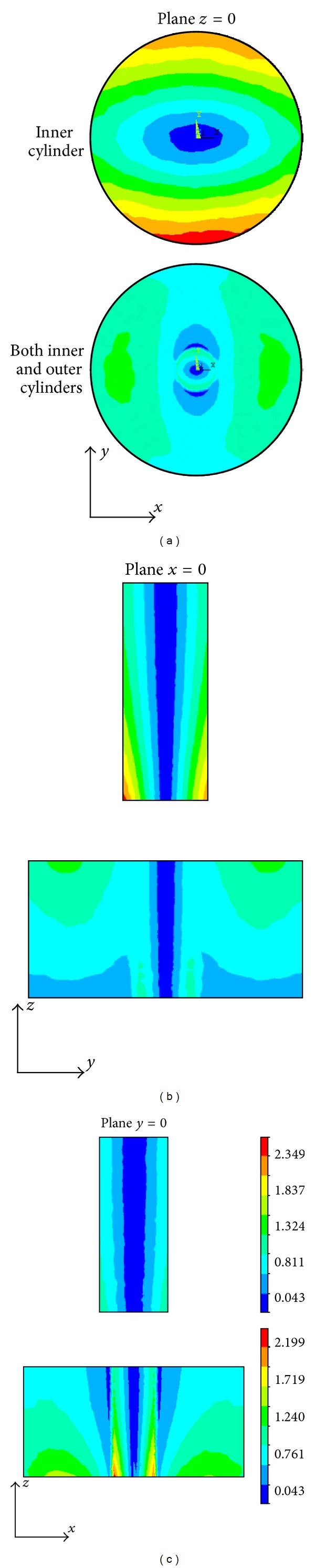
The distribution of Lorentz force density in the inner and outer cylinders on the planes *z* = 0, *x* = 0, and *y* = 0. The unit of Lorentz force density is Newton/m^3^ (N/m^3^).

Figure 8 shows the Lorentz force densities evoked in the conductive cylinders. Generally, the Lorentz force densities of the points neighboring to the boundary are larger than those in deep parts of the conductor because of the “skin effect” of the eddy current density. The force densities in the inner cylinder on the *x* direction are vastly smaller than those on the *y* direction. Since the conductor and the magnetic field are basically symmetric, only the anisotropic conductivity contributes to the asymmetric distribution of the Lorentz force density.

**Figure 9:**
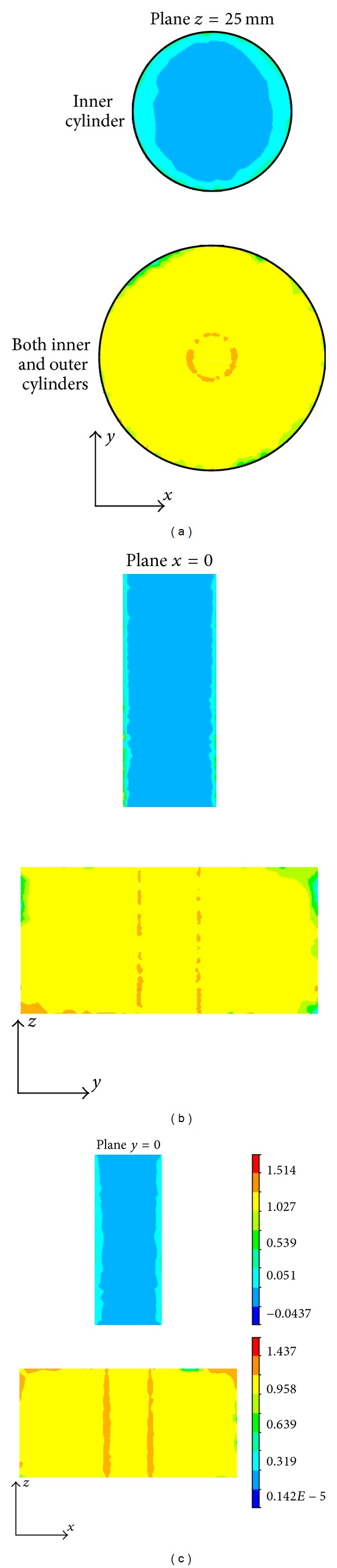
Sound source density distribution in the inner cylinder and both the inner and outer cylinders on the planes *z* = 25 mm, *x* = 0, and *y* = 0. The unit of the boundary source density is Pa/m, and that of the homogeneous source density is Pa/m^2^.

The MAT-MI source densities in the homogeneous anisotropic conducting medium and on the boundary layer are shown in Figure 9. The boundary source densities are closely associated with the magnetic field **B**, the electric field **E**, and the surface orientation. MAT-MI induces high source densities on the boundary of inner cylinder, which is in the deep part of the models. This indicates that MAT-MI can excite deep materials and therefore have a potential to image deep structures of biological tissues.

**Figure 10:**
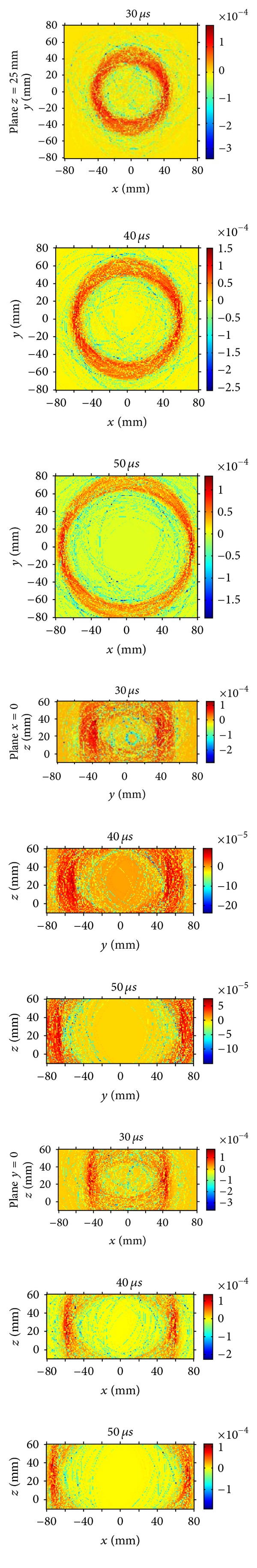
Sound pressure on the planes *z* = 25* *mm, *x* = 0, and *y* = 0 at times *t* = 20, 30, 40, and 50 *μ*s. The unit of sound pressure is Pascal (Pa).

Multiplying the boundary source density with the area of the surfaces and the source density in the homogeneous anisotropic conducting media with the volume of the tetrahedral elements and assuming that there is no sound reflection between the water and air, we compute the sound pressure in the two-layer coaxial cylinder and water medium. The pressure on the planes *z* = 25 mm, *x* = 0, and *y* = 0 is shown in Figure 10. 

**Figure 11:**
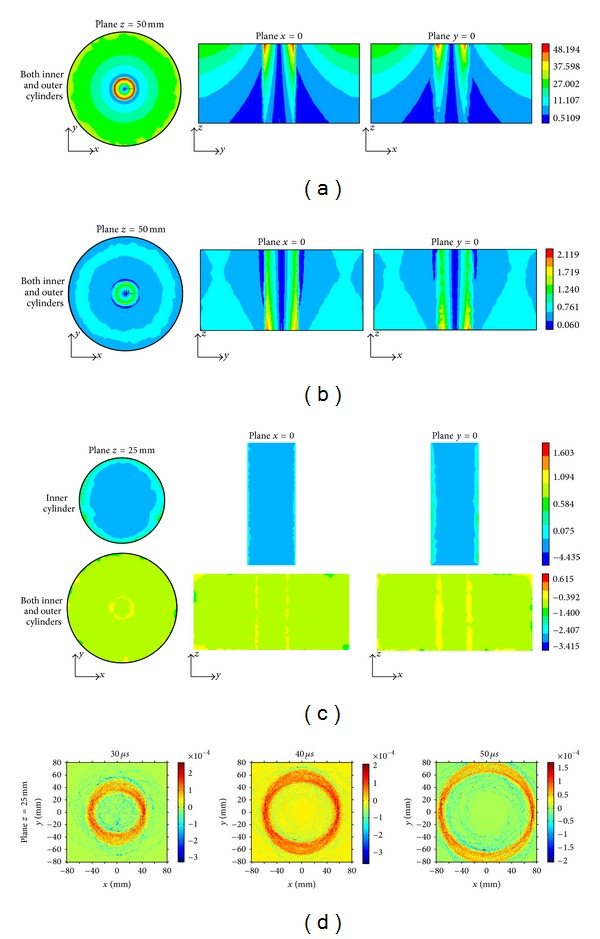
Numerical simulations on the cylindrical conductive models with isotropic conductivities. (a) Eddy current density, (b) Lorentz force density, (c) sound source density on the planes *z* = 25 mm, *x* = 0, and *y* = 0, and (d) sound pressure on the plane *z* = 25 mm at time *t* = 20, 30, 40, and 50 *μ*s. The units of eddy current density, Lorentz force density, boundary source density, homogeneous source density, and sound pressure are, respectively, Ampere/m^2^ (A/m^2^), Newton/m^3^ (N/m^3^), Pascal/m (Pa/m), Pascal/m^2^ (Pa/m^2^), and Pascal (Pa).

From Figure 11(a), we can see that the distribution of the eddy current density is symmetric because of the symmetry of the model, magnetic field, and the isotropic conductivity. Comparing Figures 11(a) and 11(b) with Figures 7 and 8, the conductivity anisotropy alters the distribution of the eddy current density and Lorentz force density in the conductor. The distribution of the sound source density in the isotropic conductive model, as shown in Figure 11(c), is similar to that in the anisotropic conductor, as shown in Figure 9. Due to the similar source densities, the sound pressure distributions for the isotropic and anisotropic conductive models are almost same, as shown in Figures 10 and 11(d).

**Figure 12:**
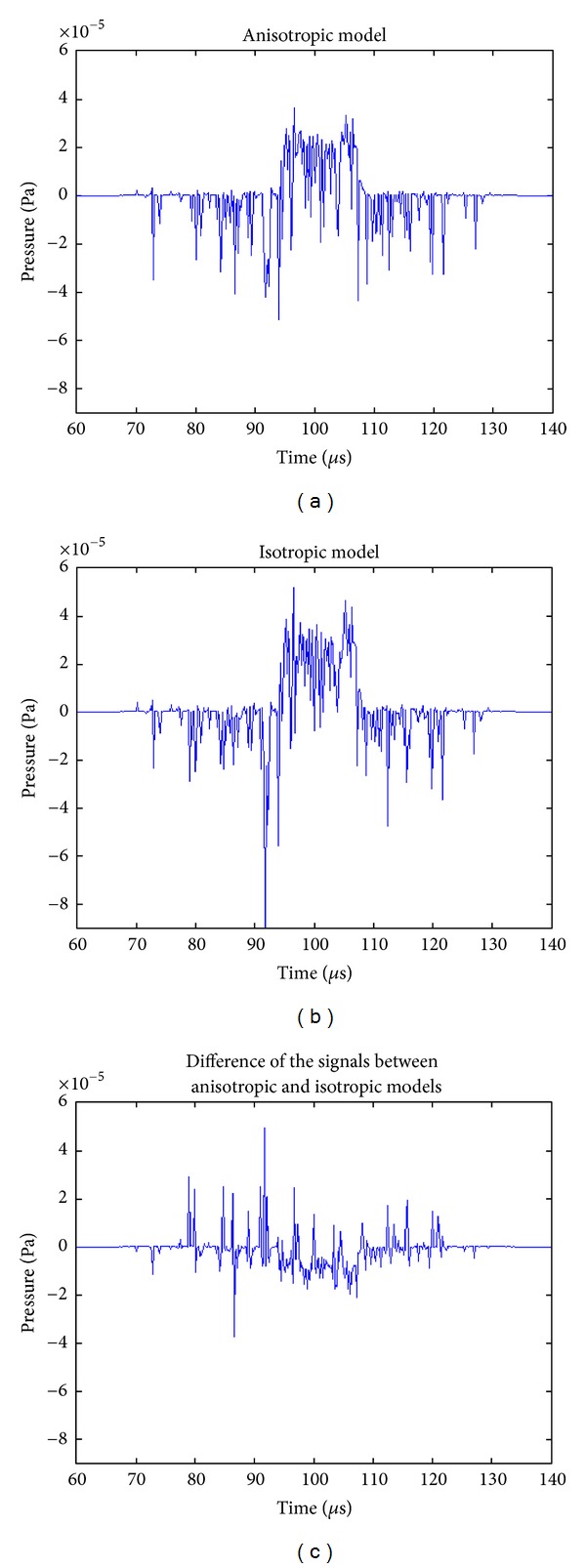
The time sequences of the acoustic signal obtained on a point whose coordinates are (0, 150, 25) mm. The unit of acoustic pressure signal is Pascal (Pa).

 We measure and compare the time sequences of the acoustic signal simulated on a point from the anisotropic and isotropic conductive models, as shown in Figure 12. The coordinates of the point are (0, 150, 25) mm. From Figure 12, we can see that the two signals have similar waveform shapes. In addition, there are some differences between the signals from the two models. Since the geometry of the model and the magnetic field are the same, the only thing contributing to the previous differences is the conductivity of the material. In other words, the different conductive properties in the two models, which are, respectively, anisotropic and isotropic conductivities, lead to such differences.

## 5. Discussions

In this simulation study, we have conducted numerical simulations on the conductive models with electrical anisotropy, the real shape coil and magnet, and calculated the MAT-MI sound source densities on the conductivity boundary. The conductivity anisotropy changes the intensity of the boundary source densities through influencing the eddy current density distribution. The effect of the electrical anisotropy in MAT-MI signal generation is not negligible. Despite of the high intensity of the boundary sources, the MAT-MI acoustic signals contain the signals radiated from the sources in the homogeneous conductive media. So, we may eliminate the impact of the boundary sources as much as possible and use weak signal detection technology to extract the useful information to reconstruct the sound sources in the homogeneous medium.

Through comparing the MAT-MI sound sources and signals from the isotropic and anisotropic conductive models, we can find that the electrical anisotropy changes the source densities and the magnitude of the acoustic pressure signals.

To investigate the MAT-MI source, the present and previous works start from the divergence of the Lorentz force density (**J** × **B**), which may cause singularity problem on the boundary, to explore the magnetoacoustic effect of biological tissues with magnetic induction. In fact, we can further perform finite element analysis of acoustic vibrations and radiations to avoid solving the divergence on the discontinuity. 

In order to study the magnetoacoustic effect of the electrical anisotropy, the present simulation conductive model is symmetric and comparatively simple. We can further create more realistic and complex breast model, including subcutaneous fat, duct system, mammographic texture, Cooper's ligaments, pectoralis muscle, skin, and abnormalities, as the breast phantom modeled for mammography [30].

Since MAT/HEI has a similar imaging principle to MAT-MI, we can further study and understand the magnetoacoustic signal generation through comparing MAT/HEI and MAT-MI. MAT injects electrical current to an object under a static magnetic field to evoke vibrations, while MAT-MI imposes time-variant magnetic field on the object under the static magnetic field to generate acoustic signals. The current injection in MAT/HEI and the magnetically induced currents in MAT-MI make a difference in the MAT/HEI and MAT-MI acoustic signals. The basic difference between MAT/HEI and MAT-MI is shown in Table 2. 

In a homogeneous isotropic conducting medium, neglecting the secondary magnetic field produced by the injected currents, the curl of the current density **J** is equal to zero in MAT [7]. Due to magnetic induction, the curl of the current density **J** in MAT-MI is associated with the electrical conductivity *σ* and the partial derivative of the magnetic flux density **B** with respect to time *t* [21]. So, there is no MAT/HEI source evoked in the homogeneous isotropic conductive domain. On the contrary, MAT-MI generates the acoustic vibrations in the whole homogeneous isotropic medium. 

On the conductivity boundary, both MAT/HEI and MAT-MI obey the same electromagnetic field boundary conditions, so we can use the same approach, as described in the Section 2.2 to solve the divergence of the Lorentz force. Roth et al. studied the MAT source and put forward that the curl of the eddy current density **J** is nonzero only at the boundary, that there is no source on the surface that is perpendicular to the applied magnetic field **B**, and that the component of the magnetic field that is perpendicular to a surface has no contribution to the source [7]. From the formula described in Table 2, for the MAT/HEI and MAT-MI boundary source density, it is clear that the intensity of the source density is zero when the dot product of the **e**_*t*′_, which is a vector on the boundary surface, and **B** is equal to zero. If we decompose **B** into three orthogonal components *B*_*n*_, *B*_*t*_, and *B*_*t*′_, **e**_*t*′_ and *B*_*n*_ are perpendicular, and the corresponding dot product is zero, that is, *B*_*n*_ contributes nothing to the source. Obviously, formula for the MAT-MI and MAT/HEI boundary sources analyzed in this paper is well consistent with the previous conclusions, and furthermore, with the analytical expression of formula, we can solve the intensities of the MAT-MI source densities on the boundary for biological tissues or phantom with arbitrary geometry.

In summary, we have created a magnet, a coil, and a two-layer coaxial cylindrical conductive model to conduct simulations for MAT-MI forward problem under conditions of inhomogeneous static magnetic field, inhomogeneous time-variant magnetic field, and conductivity anisotropy. We have also quantitatively computed the MAT-MI boundary source densities and the source densities inside the homogeneous conducting medium. To the best of our knowledge, it is the first time that MAT-MI forward problem is solved in a conductive specimen with conductivity anisotropy together with a permanent magnet. The present models and the simulation approach based on the finite element method enable us to investigate MAT-MI signal generation in a more practical simulation environment, such as arbitrary geometric configurations of anisotropic and isotropic conductive model, inhomogeneous static magnetic field produced by a permanent magnet, and various types of time-variant magnetic field generated by a coil or coil set, and so on. 

## Figures and Tables

**Table 1:** Numerical studies on MAT-MI.

	Time invariant magnetic field	Static magnetic field	Model		Sound source
			Conductivity	Structure
Xu and He 2005 [11]	H	H	H and Iso	A sphere	Analytical solution
Li et al. 2006 [18] Li et al. 2007 [19]	H	H	Inh and Iso	A two-layer concentric sphere	Numerical solution based on magnetic vector potential and electrical scalar potential method
Brinker and Roth 2008 [20]	Inh	H	H and Ani	A uniform sheet of tissue	Analytical and numerical solutions based on magnetic vector and electrical scalar potential method
Li et al. 2009 [21]	Inh	H	Inh and Iso	Two-layer eccentric spheres; a circular coil	Numerical solution based on finite element method
Li and He 2010 [22]	Inh	H	Inh and Iso	2D conductive sample; coil group	Numerical solution based on finite element method
Li 2010 [23]	Inh	H	Inh and Iso	Human breast and tumors; a circular coil	Analytical solution for homogeneous medium and conductivity boundary; numerical solution based on finite element analysis
Zhou et al. 2011 [24]	Inh	H	Inh and Iso	Breast tumor model; a circular coil	Analytical and numerical solutions using finite element method

*H denotes homogeneous; Inh denotes inhomogeneous; Iso denotes isotropic; Ani denotes anisotropic.

**Table 2:** Comparisons of MAT and MAT-MI sound generation in an inhomogeneous isotropic conductive medium.

	Boundary conditions	Sound sources	
		Homogeneous	Boundary
MAT/HEI	*E*_1t_ = *E*_2t_, *J*_1n_ = *J*_2n_	(∇×**J**) = 0	∫_*V*_∇·(**J** × **B**)*dV* = **e**_*t*′_((σ_2_ − σ_1_)*E*_1t_ *dS*) · **B**
MAT-MI	*B*_1n_ = *B*_2n_, *H*_1t_ = *H*_2t_	$\left(\nabla \times J\right)=-\sigma \frac{\partial B_{1}}{\partial t}$	
